# Changes in the network structure of mental health after a multicomponent positive psychology intervention in adolescents: A moderated network analysis

**DOI:** 10.1111/aphw.12363

**Published:** 2022-04-24

**Authors:** Claudia Tejada‐Gallardo, Ana Blasco‐Belled, Carles Alsinet

**Affiliations:** ^1^ Universitat de Lleida Lleida Spain; ^2^ Universitat de Girona Girona Spain

**Keywords:** mental health, multicomponent positive interventions, network analysis, psychological distress, well‐being

## Abstract

The effectiveness of multicomponent positive psychology interventions (MPPIs) on adolescents' mental health has been studied with the use of standard procedures throughout the scientific literature. However, little is known about the potential mechanisms underlying the network structure of mental health following the dual‐factor model after an MPPI. We relied on network analysis to explore the reorganization of the connections between mental health indicators after a school‐based MPPI. Adolescents from two high schools in Spain were randomly allocated to the 6‐week intervention group (*n* = 85) or to the control group (*n* = 135). Network analysis showed that the relations between the two differentiated network dimensions of mental health (i.e. well‐being and psychological distress) changed after the intervention. Unlike control participants, emotional well‐being was negatively associated with depression and stress, while psychological well‐being was positively related to stress after the intervention. The present study supports the viability of the network approach in analyzing the connections between mental health indicators as defined by the dual‐factor model and the contribution of MPPIs to change the complex pattern of relations between the dimensions of well‐being and psychological distress.

## INTRODUCTION

Can school‐based multicomponent positive psychology interventions (MPPIs) change the network structure of mental health? If so, to what extent? Positive psychology interventions (PPIs) originated as scientifically based interventions that focus on strengthening positive emotions, thoughts, and behaviors through activities that can be easily implemented in daily routines (Schotanus‐Dijkstra et al., 2015). Compared to PPIs, MPPIs are composed of a variety of exercises targeting two or more theoretically relevant well‐being components (e.g. gratitude and optimism). A meta‐analytic review of school‐based MPPIs of adolescents showed small to moderate effects immediately after the intervention and in the long term on well‐being and psychological distress (Tejada‐Gallardo et al., 2020). MPPIs are framed under the fledgling science of positive psychology, which involves the study of optimal human functioning that can be pursued through hedonic well‐being—feeling good—or eudaimonic well‐being—functioning well in life. Although this positive approach to well‐being has been widely accepted by the scientific community, the conventional view of mental health as the mere absence of psychological symptomatology and negative outcomes is still widespread (Seligman & Csikszentmihalyi, 2000).

Despite the deep‐rooted medical approach of illness prevention, there is an increased recognition of the conceptualization of complete mental health as defined by the dual‐factor model, which describes *positive* mental health as the presence of well‐being and the absence of psychopathology (Keyes, 2009). Given the increasing importance of promoting positive aspects of mental health in research and policy agendas (Barry, 2009), it is of interest to understand in a more detailed manner how the network structure of adolescents' mental health, based on the dual‐factor model, can change after a school‐based MPPI. Such research would illuminate the potential mechanisms underlying the efficacy of these interventions and identify the best routes for driving positive changes in mental health.

### The dual‐factor model of mental health

The traditional conceptualization of mental health as the absence of psychopathology in psychology and psychiatry illustrated a bipolar relation between mental health and mental illness in which individuals are either mentally ill or mentally healthy (Keyes, 2005; World Health Organization, 2004). But the growing number of studies investigating well‐being incorporated into the concept of positive mental health aspects like positive emotions, character strengths, optimal psychological functioning, and positive relations, among other factors (Fredrickson, 2001; Keyes, 2009; Park & Peterson, 2009). The integration of well‐being and psychological distress as related but empirically distinct indicators of mental health is widely supported in general and clinical populations (Iasiello & Van Agteren, 2020; Trompetter et al., 2017; Westerhof & Keyes, 2010), the central arguments being that psychopathology can co‐occur with high levels of subjective well‐being (Greenspoon & Saklofske, 2001) and that high levels of well‐being can protect against the development of mental illness (Keyes et al., 2010). This suggests that well‐being and psychopathology are not opposite poles of a single continuum and that individuals can experience high levels of mental health despite instances of mental illness (Suldo & Shaffer, 2008).

But why is it important to introduce the dual‐factor model of mental health in adolescents? During adolescence, emotional inclinations often stabilize and negative inclinations can develop into stable and pathological states of, for instance, depression and anxiety (Derdikman‐Eiron et al., 2013). During the transition from adolescence to adulthood, there is a high prevalence of adolescents suffering from mental diseases, most of which also develop during this stage, especially depression (Pearson & Wilkinson, 2013). Although psychological distress is a prevailing concern during this life stage, it has been demonstrated that high levels of well‐being are related to decreased levels of depression in adolescents (Doll, 2008; Tejada‐Gallardo et al., 2021). For this reason, targeting multiple domains of positive functioning through MPPIs would be a promising pathway for increasing well‐being and reducing psychological distress (Tejada‐Gallardo et al., 2020). Also, the dual‐factor can serve as a model to embrace the complexity of mental health to design interventions aimed at targeting the two indicators of mental health in efforts to promote the optimal psychological functioning of adolescents.

### School‐based MPPIs for promoting adolescents' mental health

Applying the dualistic view of mental health carries the need to test whether psychological interventions have an impact on both indicators. Applied research has shown that the dual‐factor model can be the basis to explain changes in well‐being and psychological distress after psychological interventions (Teismann et al., 2018; Trompetter et al., 2017), and a recent meta‐analysis has demonstrated that MPPIs are among the most effective interventions to promote well‐being across clinical and non‐clinical populations (van Agteren et al., 2021). In MPPIs, multiple domains of positive functioning are targeted, this includes relevant well‐being components such as gratitude practices that enhance past positive emotions and reduces negative thought patterns or optimism practices that increase positive expectations regarding the future and prevent depression (Miller & Nickerson, 2008). Given the effectiveness of school‐based MPPIs (Tejada‐Gallardo et al., 2020), transition and adaptation to young adult life is healthier when adolescents experience positive mental health and relatively minor symptoms of psychopathology. Hence, MPPIs can be considered as effective mental health promotion programs to track the evolution of well‐being and psychological distress symptoms in adolescents.

In order to successfully continue supporting the effectiveness of school‐based MPPIs, further research is needed to scrutinize their effects on mental health. Studies investigating the effectiveness of positive psychology programs have typically used mean differences and effect sizes (e.g. Tejada‐Gallardo et al., 2020). These standard procedures to assess the efficacy of treatments or interventions may not reveal deep differences in how interventions work (Cheung & Slavin, 2016), although they align with existing research that has illuminated their efficacy. However, whether and how the structure and dimensions of mental health change, as defined by the dual‐factor model, after the implementation of an MPPI remain understudied. To address this gap, the network approach can be applied to the study of MPPIs and to better understand the underlying complexity of adolescents' mental health.

### How can the network approach contribute to the study of the dual‐factor model of mental health?

A growing number of empirical studies using the network approach have emerged over the last years (Fried & Cramer, 2017). In the network approach, psychological constructs are defined as systems of interacting components (e.g. behaviors, abilities, symptoms; Barabási, 2011). In the case of psychopathology, the mutual interactions between symptoms explain the emergence of the mental illness (Borsboom, 2017). For example, major depression arises because having sleep problems lead to being tired and to concentration problems (Cramer et al., 2016); rather than being caused by an underlying brain disorder, major depression might be the cause of the co‐occurrence of symptoms. The application of the network approach has been proposed as a promising and fruitful line of inquiry into the science of well‐being (Fabian, 2021). Psychological well‐being has been recently approached as a network model to examine its structure; despite not replicating the premises of the psychological well‐being theory, the findings provide an initial step to conceptualize well‐being constructs as network systems (Blasco‐Belled & Alsinet, 2022).

Network models provide user friendly graphs to visualize the data, in which variables are represented as nodes, and the relations between the variables are represented as edges (Epskamp et al., 2018). The main aim of network analysis is to investigate the structure of relations between the network components. Positive edges indicate that an increase in activation of that edge is related to an increase in activation of the second edge, whereas negative edges indicate that an increase in activation of one edge is related to a decrease of activation of the other edge (Rodebaugh et al., 2018). Following the dual‐factor model, mental health in the present study would be considered as a network of two interacting dimensions (well‐being and psychological distress), each one including three subscales (leading to a total of six nodes): emotional, psychological, and social well‐being as indicators of the well‐being dimension, and depression, anxiety, and stress as indicators of the psychological distress dimension.

It could be hypothesized that the mutual positive relation between emotional, social, and psychological well‐being will give rise to the dimension of well‐being, while the mutual positive relation of depression with anxiety and stress are hypothesized to form the dimension of psychological distress. How well‐being and psychological distress interact with each other will characterize the ultimate emergence of mental health as defined by the dual‐factor model. Specific analysis of the interactions between nodes from the two dimensions after an MPPI can open new possibilities to explore the network structure of mental health over time beyond the dualistic view of the absence/presence of psychopathology and identify potential mechanisms underlying the efficacy of MPPIs.

## THE PRESENT STUDY

The goal of the present study was to analyze to what extent an MPPI can change the dynamic network structure of mental health as conceptualized by the dual‐factor model in two samples of adolescents (intervention and control). To this end, two outcomes were assessed: well‐being (emotional, social, and psychological) and psychological distress (depression, anxiety, and stress). We have no previous evidence on network models of well‐being to sustain our expected hypotheses; however, research on MPPIs showed higher effects on subjective and psychological well‐being at post‐test compared to follow‐up assessments. As such, and based on previous research on MPPIs and mental health, we expect to find a more connected structure in the intervention group at post‐test measurement, implying more robust positive connections *within* each of the two dimensions, and potentially more negative connections *between* the two dimensions. This would indicate that the MPPI would have had an effect on improving well‐being and reducing psychological distress.

## METHODS

### Participants and procedure

Participants were 220 adolescents^1^ from two high schools in western Catalonia (Spain) who were randomly allocated to the intervention group (*n* = 85) or control group (*n* = 135). The overall mean age was 14.98 years (*SD* = 0.62). Further demographic data are presented in Table 1. The responses of the intervention participants were only included in the analyses if they attended at least four of the six program sessions. Hence, from the initial sample, only 79 participants from the intervention group (51.8% females) and 134 from the control group (44.4% females) were retained for further statistical analyses. Informed consent signed by parents or tutors was required from all participants, and they were informed that they could withdraw from the study at any time. A total of six positive psychology sessions were offered from October 2019 to December 2019. The Get to Know Me+ intervention was implemented by two PhD psychology students. Measures were taken 1 week before the intervention (pretest), 1 week after the intervention (post‐test), and 2 months after the intervention ended (follow‐up). The present study was approved by the University Ethics Committee under the code CEIC‐2157.

**TABLE 1:** Sample demographics reported at baseline assessment

	Intervention (*N* = 85)		Control (*N* = 135)	
Demographic	*N*	%	*N*	%
Gender				
Female	44	51.8	60	44.4
Male	41	48.2	73	54.1
Other	0	0	2	1.5
Ethnicity				
Hispanic, Latino, or other	6	7.0	8	5.9
Spanish origin	69	81.2	118	87.4
Not Hispanic	10	11.8	9	6.7
Socioeconomic status				
Low	16	18.8	36	26.7
Average	56	65.9	86	63.7
High	13	15.3	13	9.6
Family composition				
Both parents together	59	69.4	111	82.2
Only one of the parents	20	23.5	24	17.8
Other family member	6	7.1	0	0

### The get to know me+ program

This intervention is a face to face 6‐week program featuring aspects with a strong connection to subjective and psychological well‐being (i.e. well‐being, character strengths, emotions, optimism, gratitude, and goal setting), based on empirical grounds identified in well‐being research (see Table S1 for session planning). The main goals of the program are (1) to enhance the well‐being of adolescents during the transition process to young adulthood; (2) to help adolescents overcome the challenges that they face personally and socially, promoting an optimal psychological functioning; and (3) to develop positive feelings toward time (past, present, and future). According to previous meta‐analysis of MPPIs, the more sessions included in these programs (at least six sessions), the more efficacious they are (Bolier et al., 2013; Tejada‐Gallardo et al., 2020). However, The Get to Know Me+ program used in this study had a limited duration of 6 weeks due to the schools' schedules. The study version of the program was designed to function as an integrated whole composed of the principles of well‐being under three modules: (1) focus on the positive emotions of the present; (2) deal with the positive emotions of the past; and (3) move forward toward the positive emotions of the future. Each session lasted 1 h and consisted of three parts with an introductory flow activity, a central activity to put in practice the principle of well‐being, and the closing of the session.

### Measures

Well‐being was assessed by the Mental Health Continuum‐Short Form (MHC‐SF; Keyes et al., 2008; Spanish adaptation of Echeverría et al., 2017). The MHC‐SF assesses emotional, social, and psychological well‐being during the previous month. This scale consists of 14 items, and respondents rate the frequency of each feeling in the past month on a 6‐point Likert scale (1 = *never*, 6 = *every day*). The following sample items are representative of each subscale: “In the past month, how often did you feel happy?” for emotional well‐being; “In the past month, how often did you feel that you had something important to contribute to society?” for social well‐being; and “In the past month, how often did you feel that you liked most parts of your personality?” for psychological well‐being. The Cronbach's *α* reliability estimates of the MHC‐SF for Time 1 were .77 for emotional well‐being, .71 for social well‐being, and .79 for psychological well‐being. For Time 2, the estimates were .86 for emotional well‐being, .80 for social well‐being, and .82 for psychological well‐being. For Time 3, they were .85 for emotional well‐being, .80 for social well‐being, and .82 for psychological well‐being.

Psychological distress was assessed by the Depression, Anxiety, and Stress Scale (DASS‐21; Lovibond & Lovibond, 1995; Spanish adaptation of Daza et al., 2002). The DASS‐21 assesses the levels of symptomatology associated with depression, anxiety, and stress during the previous week. This scale consists of 21 items, and responses are based on a 4‐point Likert scale (0 = *did not apply to me at all*, 3 = *applied to me very much or most of the time*). The following sample items are representative of each subscale: over the past week, “I could not seem to experience any positive feeling at all” for depression; “I was worried about situations in which I might panic and make a fool of myself” for anxiety; and “I find it hard to wind down” for stress. The Cronbach's *α* reliability estimates of the DASS‐21 for Time 1 were .84 for depression, .73 for anxiety, and .76 for stress. For Time 2, the estimates were .88 for depression, .83 for anxiety, and .79 for stress. For Time 3, they were .89 for depression, .83 for anxiety, and .84 for stress.

### Statistical analyses

#### Moderated network estimation

To estimate group differences in network models applying the Moderated Network Model (MNM) approach, we used the *mgm* R‐package version 1.2‐9 (Haslbeck & Waldorp, 2020). Visualization of networks is accomplished with nodes (variables) and edges (connections), in which the width of edges indicates the strength of the connections. In our case, edges represented conditional dependencies between two variables after controlling for all other variables of the network. We used Gaussian Graphical Models (GGM), which represent the unique associations between two variables after conditioning on the rest of variables of the network (Epskamp et al., 2018). This means that a negative connection between emotional well‐being and depression, for instance, can be taken as an indication that an individual scoring high on emotional well‐being will tend to score low on depression and that this cannot be explained by other variables.

The MNM approach enables the fitting of networks using variables of mixed types (Mixed Graphical Models; MGM), in which one variable acts as a moderator of the pairwise interaction between two nodes. We fitted a moderated MGM for each timepoint measurement (pre, post, and follow‐up) that included a grouping variable with two categories (moderator) and six continuous variables corresponding to measures of mental health and psychological distress. The grouping variable (i.e. control or intervention group) was introduced as a categorial moderator, allowing a comparison of group differences by conditioning on the moderator. For instance, this answered the question, “*Does the relationship between emotional well‐being and depression differ between the control and intervention groups?”* or “*Do differences in the relationship between emotional well‐being and depression depend on allocation to the control or intervention group?”* The moderated networks were conditioned on the grouping variable using the function *condition* of the *mgm* R‐package (Haslbeck & Waldorp, 2020). We specified the values of the moderator to represent the control (1) and experimental (2) groups for the three timepoints. *mgm* implements a regularization parameter in the ℓ1‐regularized nodewise regression algorithm. We selected the regularization parameter with cross‐validation with a hyperparameter of *γ* = 0.25 and AND‐rule to combine estimates across nodewise regressions. Cross‐validation is a less conservative model selection preferable with small samples because it has greater sensitivity in revealing results but at the risk of lower specificity; that is, there is a higher probability of identifying true edges in the network but a higher chance of including false edges (Epskamp & Fried, 2018).

#### Stability of edge‐estimates

To test the stability of the estimated parameters in the MNMs, we used the function *resample* of the *mgm* R‐package (Haslbeck & Waldorp, 2020), which obtains empirical sampling distributions using the nonparametric bootstrap—in our study, we applied 50 bootstrap samples. The function *plotRes* returns a plot of the bootstrapped sampling distribution of each 2‐way and 3‐way interaction. Small variance in the sampling distribution suggests that the network is stable, and nonzero values with 95% confidence intervals that exclude zero indicate likelihood of moderation effects in the network model (see Haslbeck et al., 2021; Haslbeck & Waldorp, 2020, for more details). To confirm the presence of moderation effects, we compared the sampling distributions with simulated data from a null model with no moderation effects (see Supplementary material). Analyses were carried out in *Rstudio* version 1.3.1093 (RStudio Team, 2020).* The code and database to reproduce the study are available at https://osf.io/sp4d7/. [Corrections made on 26 April 2022, after first online publication: The preceding URL has been updated in this version.]

## RESULTS

### Comparative analyses

Independent samples *t*‐tests comparing intervention and control groups were run at baseline, post‐test, and follow‐up. Compared to the control group, the intervention group showed higher anxiety at baseline (*t*[211] = −0.17, *p* < .05). No significant differences were reported at post‐test and follow‐up assessments, which might suggest that the intervention could have reduced the levels of anxiety in the intervention group.^2^

### Network analysis

#### Moderated network models

**FIGURE 1:**
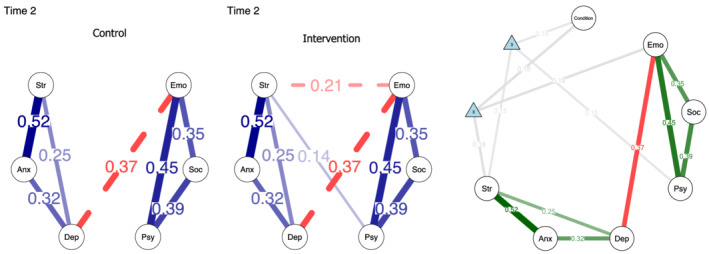
In the left panel, moderated network models at Time 2, separately presented by groups. In the right panel, factor graph represents the 3‐way interactions (moderations) as factor nodes and pairwise interactions as edges between variables at Time 2. *Note*. Soc = Social well‐being; Psy = Psychological well‐being; Emo = Emotional well‐being; Dep = Depression; Anx = Anxiety; Str = Stress

The visualizations of the MNMs for each timepoint measurement are presented in Figure S1. Blue edges represent positive linear relations, red edges represent negative linear relations, and gray edges represent relations related to the moderator. Two dimensions were differentiated in the networks, corresponding to well‐being and psychological distress. Moderation (3‐pairwise) effects appeared only at Time 2 involving the variables of stress, emotional well‐being, and psychological well‐being (see Figure 1). Unlike the control group, there was a negative association between emotional well‐being and stress and a positive relation between psychological well‐being and stress in the intervention group. This suggests that group condition moderated the effect of stress with emotional and psychological well‐being. Of note, the negative (and positive) relation of depression to psychological well‐being (and stress) vanished over time, apparently not due to the intervention effects.

**FIGURE 2:**
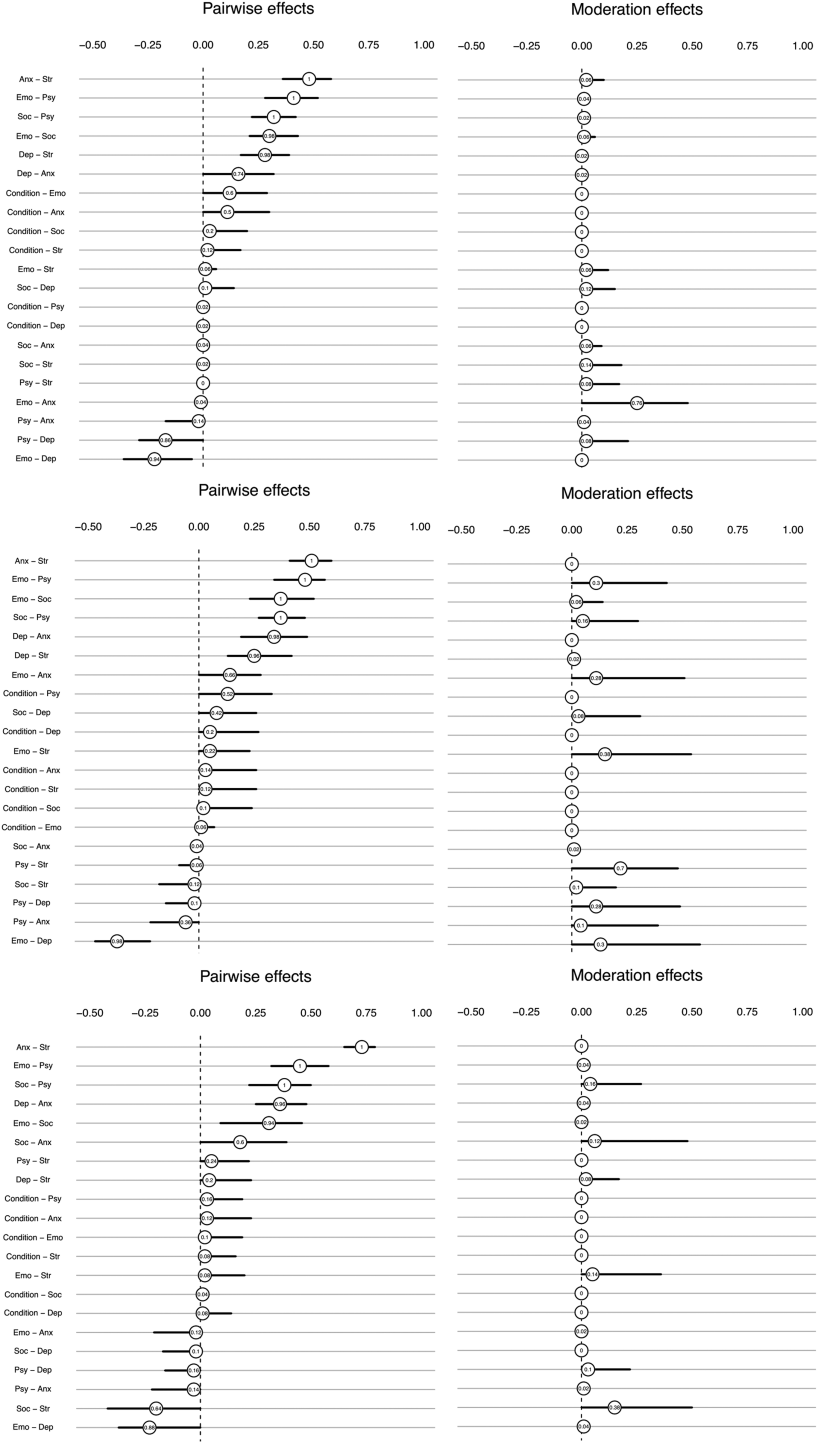
Bootstrapped sampling distributions at Time 1, 2, and 3 (in order)

Figure 2 presents the bootstrapped sampling distributions of the MNMs. The left plot shows the pairwise effects and the right plot the moderation effects. Values represent the proportion of bootstrap samples in which a given parameter was estimated as nonzero, and the lines show the 5% and 95% quantiles. The placement of the values indicates the mean of the sampling distributions. Pairwise and moderation effects were reasonably stable over time, and moderation effects were substantially stronger at Time 2, as several values were far from zero. To ensure evidence of the moderation effects, we compared our data model (moderation effects are present) against a null model (moderation effects are absent). We simulated data with a matched sample size from a standard pairwise model in which no moderation effects were present (Figure S2). The bootstrap sampling distributions from the null MNM (representing Time 2) were generally close to zero (Figure S3), reinforcing that the presence of moderation effects was due to the intervention and not sampling variation.

## DISCUSSION

The present research analyzed changes in the network structure of mental health based on the dual‐factor model after a school‐based MPPI (Get to Know Me+) to gain a more thorough understanding of the potential mechanisms underlying the efficacy of these interventions. Previous research examined the effectiveness of MPPIs in adolescents employing standard procedures (i.e. mean differences and effect sizes; Tejada‐Gallardo et al., 2020), which preclude identifying changes in the structure and dynamics of adolescents' mental health after a school‐based MPPI. Investigating the relations between the individual variables that form the complex system of mental health, this means analyzing how indicators of well‐being and psychological distress relate to each other, might be an initial step toward the identification of dynamics that cause and hold a state of positive mental health. To achieve this purpose, the network approach—more specifically, the MNM—was applied to compare group changes in the network structure of mental health at post‐test and follow‐up assessment. Differences across groups were found after the intervention, addressed in more detail in the remaining discussion.

### Changes in the network structure of mental health after an MPPI

The structure of mental health in both groups was composed of two differentiated dimensions that resemble the theoretical underpinnings of the dual‐factor model (Keyes, 2009): well‐being (emotional, social, and psychological) and psychological distress (depression, anxiety, and stress). The dual‐factor model has traditionally conceptualized mental health as an underlying unobservable variable that characterizes high levels of well‐being and low levels of psychological distress. Nevertheless, the network model of mental health estimated in this study allowed to observe in more detail the links between these two dimensions. Psychological distress and well‐being were bridged by negative connections of depression with emotional and psychological well‐being prior to the intervention. However, differences in the connections of these indicators across groups were found after the implementation of the MPPI.

First, there was an increase in the coefficients, suggesting that the structure of the dimensions was more robust at Time 2 than at Time 1. Second, compared to the control group, stress was associated positively with psychological well‐being and negatively with emotional well‐being in the intervention group. Nevertheless, the moderation effects of the MPPI vanished over time, and at follow‐up assessment both groups reported the same network structure of mental health. At this point, the connections of stress with emotional and psychological well‐being were no longer present, nor was the positive connection between depression and stress, indicating that the longitudinal effects of the MPPI in the network structure of mental health were weaker than at post‐test, akin to the majority of positive evidence‐based psychological interventions (Carr et al., 2020).

These findings highlight the role of stress and different reasons might explain the emergence of this indicator as central to the network of mental health. First, the dimension of stress from the DASS‐21 is considered the most different subscale compared to depression and anxiety, and calculating specific scores on this subscale might be erroneous because it is best conceived as a measure of general distress in adolescent samples (Jovanović et al., 2019). Because the network approach understands the network of mental health as a system, it can help overcome the limitation of construct validity of the subscale to better understand its dimensionality. The fact that stress was allowed to interact with all the components that form the system facilitated stress to emerge as an important component of mental health, which would not be the case using a latent factor analysis.

Second, stress is a psychological symptom that can either be seen as a barrier or a motivator to establish goals in adolescents (Gutowski et al., 2018). Adolescents usually report higher levels of stress compared to children (Stroud et al., 2009) and adults (American Psychological Association Stress Report, 2014), which may be a consequence of the high demands that they face during this transitionary stage. For instance, life purpose has been considered a developmental asset and an indicator of thriving (Bronk & Finch, 2010), notwithstanding that, at the same time, it increases adolescents' perceived levels of stress (Hughes, 2006). This fits within the scope of our research and demonstrates the complex role of stress in connecting with hedonic and eudaimonic aspects of well‐being. Having purpose in life is a correlate of nearly every component of well‐being (Bronk, 2014), but being unable or unwilling to find it can generate a sense of meaningless, and living without meaning, goals, or values can subsequently trigger psychological distress (Frankl, 1959). Similarly, identifying and pursuing one's purpose and meaning in life can increase the levels of stress due to the generally (socially) imposed need to achieve them (Hughes, 2006).

The convergence of our findings should be interpreted in the context of adolescence. On the one hand, the relevance of experiencing positive emotions and pleasant moments in daily life is well documented (Kahneman & Deaton, 2010). To be more specific, positive emotions can boost the activation of social and personal resources (Fredrickson, 1998) and promote emotional well‐being (Fredrickson & Joiner, 2018). With reference to our intervention, the positive emotions evoked by hedonic activities during the MPPI may have enriched the thought‐action repertories of adolescents to build useful personal resources. These resources can serve as a means of meeting the psychological demands on adolescents during eudaimonic activities, such as visualizing their future. On the other hand, although engaging in positive activities that boost psychological well‐being may help in identifying and pursuing adolescents' purpose in life, attempting to achieve important goals can also have counterproductive effects. In fact, important challenges in one's life can be appraised as threats and can increase perceived stress levels (e.g. goals ➔ threats ➔ stress; Hughes, 2006).

The Get to Know Me+ program covered hedonic and eudaimonic components of well‐being (e.g. pleasure, life meaning, or positive relations), following previous studies that reinforced the idea of activating both routes to well‐being in positive interventions (e.g. Blanco et al., 2020; Burckhardt et al., 2016). Considering the results, our MPPI was associated with positive emotions (emotional well‐being) immediately after the intervention, which may have buffered the stress evoked when working through eudaimonic activities like establishing meaningful goals or finding ways to develop strengths. These assumptions align with previous work on the predictive role of positive affect on eudaimonic well‐being (Garcia & Siddiqui, 2009; Martela et al., 2018). In this view, school‐based MPPIs can help promote relevant aspects of mental health, increase momentary positive affectivity and manage stressful symptoms that arise during the adolescent developmental stage.

The present study contributes to understand in more specific detail the potential pathways that maintain a state of high well‐being, which can serve to formulate initial hypotheses about which components of mental health can be potential targets for future psychological interventions. On the one hand, focusing on single nodes like emotional well‐being by increasing positive, hedonic emotions might be used to alleviate the levels of depression and stress. On the other hand, it would be possible to target the link between two nodes. For instance, future interventions might consider regulating the features of eudaimonic activities (dose, intensity, or frequency) to reduce the positive association among psychological well‐being and stress in adolescents.

### Constraints on generality

Our findings provide evidence of the changes in the network structure of mental health after an MPPI in adolescents attending formal education (i.e. high schools). More specifically, the sample targeted late adolescence (10th grade), a transitional period of this life stage when adolescents must face various demands in life. We believe the results will be reproducible with adolescents presenting similar characteristics and engaging in the same (or akin) intervention program. Cross‐validity and direct replication would test the changes in the network of mental health using a comparable sample but in other countries (i.e. cross‐cultural studies). However, we do not have evidence that the same findings will occur in other age group samples (e.g. primary school children or university students) or in adolescents with different characteristics as the presented above because network analysis studies that investigate mental health after an intervention are still scarce. We have no reason to believe that the results depend on other characteristics of participants, materials, or context.

### Limitations

The present study is not without limitations. First, the design of the study employed a randomized‐controlled trial in which adolescents were randomly allocated to the intervention group. In this situation, adolescents do not normally engage actively in the activities proposed and, consequently, are not likely to make deliberate efforts to obtain substantial results (Sheldon & Lyubomirsky, 2019). Hence, well‐being interventions are likely to be more effective among individuals who participate voluntarily. It may be of interest to conduct future studies only with adolescents genuinely willing and motivated to participate in the intervention. Additionally, training in well‐being techniques is likely to be more effective among people in need of improvement (Bergsma et al., 2020). We did not initially screen adolescents' levels of well‐being, and, as a result, a percentage of adolescents allocated in the intervention group may have already been experiencing high levels of mental health (i.e. flourishing). In this case, improving their levels of well‐being would be a challenging task (Sarriera et al., 2017). In addition, the sample size of the study was rather small and future network analysis studies should include larger samples in order to accurately estimate a hypothesized network. Finally, because the network structure represents undirected relations, it is not possible to clarify directionality in them, and despite the longitudinal design of the study, these findings do not imply causality. Thus, future work may wish to investigate network structure using momentary assessment to capture temporal and contemporaneous interactions between indicators. Given that the results are data‐dependent, caution is needed in the interpretation of the potential implications.

### Conclusions

Applying the network approach can be a valuable step to studying the patterns of changes in adolescents' mental health after a school‐based MPPI. Our findings provide support for the conceptual premise that mental health is an interactive and dynamic network of well‐being and psychological distress. The introduction of the network approach offers the possibility of exploring patterns of change in the network structure of adolescents' mental health from pre‐ to post‐intervention. The results highlighted changes immediately after the intervention in relation to hedonic and eudaimonic aspects of well‐being. Because this is the first study attempting to analyze patterns of change in the network structure of mental health after a school‐based MPPI in adolescents, further research is needed on the topic.

## CONFLICT OF INTEREST

The authors declare that they have no conflict of interest.

## ETHICS STATEMENT

This study was approved by the Lleida University Ethics Committee under the code CEIC‐2157.

## INFORMED CONSENT

Participant's parent/guardian provided written informed consent before participating in this study.

## Supporting information


**Table S1.** *Summary of Intervention Contents*
**Figure S1.** Moderated network models for Time 1, 2 and 3, separately presented by groups
*Note*. Emo = Emotional well‐being; Soc = Social well‐being; Psy = Psychological well‐being; Dep = Depression; Anx = Anxiety; Str = Stress.
**Figure S2.** Factor graphs of the MNM at Time 1, 2 and 3, respectively. Factor graphs allow a powerful visualization of moderated networks by including 3‐way and 2‐way interactions as additional nodes. In the left panel, white nodes represent the network variables, red squares represent pairwise interactions, and blue triangles represent 3‐way interactions (moderations). In the right panel, 3‐way interactions (moderations) are presented as factor nodes and pairwise interactions as edges between variables. Blue edges represent positive linear relationships, red edges represent negative linear relationships, and grey edges represent relationships related to the moderator.
*Note*. Abbreviations are described in Figure S1. Condition is the grouping variable (control or experimental group).

## Data Availability

The data and materials supporting the analyses presented in this manuscript can be found at https://osf.io/sp4d7/

## ENDNOTES

^1^
Post hoc power analysis was calculated using G*Power (v. 3.1.9.7; Faul et al., 2009) to detect small (i.e. 0.20) effect size with the level of alpha = .05 and *n* = 220 (the sample size at pretest). The results indicated a power of 0.92. Likewise, the power was 0.91 for the sample size at post‐test (*n* = 213), using the same effect size and alpha parameters.

^2^
All results were also analyzed controlling for age and no significant differences emerged
