# Supplementary material for: Changes in the network structure of mental health after a multicomponent positive psychology intervention in adolescents: A moderated network analysis
Source: Appl Psychol Health Well Being. 2022 Apr 24;14(3):987–1003. doi: 10.1111/aphw.12363 (PMC9545719; doi:10.1111/aphw.12363)
Supplement: Supplementary file 1 — Table S1. Summary of Intervention Contents Figure S1. Moderated network models for Time 1, 2 and 3, separately presented by groups Note. Emo = Emotional well‐being; Soc = Social well‐being; Psy = Psychological well‐being; Dep = Depression; Anx = Anxiety; Str = Stress. Figure S2. Factor graphs of the MNM at Time 1, 2 and 3, respectively. Factor graphs allow a powerful visualization of moderated networks by including 3‐way and 2‐way interactions as additional nodes. In the left panel, white nodes represent the network variables, red squares represent pairwise interactions, and blue triangles represent 3‐way interactions (moderations). In the right panel, 3‐way interactions (moderations) are presented as factor nodes and pairwise interactions as edges between variables. Blue edges represent positive linear relationships, red edges represent negative linear relationships, and grey edges represent relationships related to the moderator. Note. Abbreviations are described in Figure S1. Condition is the grouping variable (control or experimental group). [file APHW-14-987-s001.docx]

**Table S1.**

*Summary of Intervention Contents*

| Modules and sessions | Session goals | | Procedure and activities |
| --- | --- | --- | --- |
| 1. Well-being | - Establish supportive group environment - Introduce students to the broad aspects of well-being (Lyubomirsky et al., 2005) and the aspects that determine it | | **Flow activity**: Mind map activity: “Which memories do you relate with your favorite fruit”?  **Central activity**: Mind map activity: “What does well-being mean to you”?  **Group discussion**: "What does well-being mean? Why is it important?”  **Closing**: Suggesting ways of increasing well-being through purposeful thoughts |
| *Focus on the positive emotions of the present* |  | |  |
| 1. Character strengths | - Define character strengths and virtues (Park et al., 2004) - Explore students’ character strengths through the VIA questionnaire and apply them to different situations | | **Flow activity**: Identify the character strengths (using a card game) of their best friend/important person  **Central activity 1**: Identify their own character strengths  **Central activity 2**: Identify and share two-character strengths of their group peers using a card game  **Central activity 3**: Select and apply their top two-character strengths in three different contexts (family, friends, school)  **Group discussion**: Discuss their previous selections with the class group  **Closing**: Discuss how character strengths are related to well-being and encourage students to use their greatest strengths |
| 1. Dealing with emotions | - Introduce the components of emotional intelligence (attention, clarity, and regulation) - Challenge negative emotions and thoughts through cognitive restructuring and describing past negative experiences (Fava, 1999) | | **Flow activity**: Identify and define each component of emotional intelligence  **Central activity**: Emotional action process. Identify a conflict situation; recognize the emotions, thoughts, and behavior that the situation evoked; finally, suggest and plan more adaptive responses to future similar situations.  **Group discussion**: Share the central activity with the class group  **Closing**: Discuss the importance of acknowledging their freedom to change and adapt their responses to distressful situations and highlight the contribution of positive emotions to well-being |
| *Turn back to the positive emotions of the past* |  | |  |
| 1. Gratitude | - Introduce gratitude and its contribution to well-being through prosocial behavior - Connect with and appreciate positive emotions - Learn to integrate actions and expressions of gratitude in their daily lives | | **Flow activity**: *The Desert Island*: “What would you bring with you to a desert island?” Importance of reminding themselves of the most important things in their life  **Central activity**: Gratitude letter  **Group discussion**: Voluntarily share the gratitude letter with the class group  **Closing**: Remind why gratitude can be important for improving well-being. Challenge students to make a gratitude visit during the week |
| *Move forward to the positive emotions of the future* | |  |  |
| 1. Optimistic thinking | - Introduce optimism and optimistic thinking - Learn strategies to establish goals and paths to reach them | | **Flow activity**: Identify and share an important memory, person, and wish  **Central activity**: My best possible self  **Group discussion**: Voluntarily share the central activity and discuss the importance of optimistic thinking with the class group  **Closing**: ﻿Remind students that hope can help them focus on positive goals for their futures and prevent feelings of helplessness through the belief that there are ways to meet those goals |
| 1. Goal setting | - Compile the activities and exercises learned through the program - Frame life in terms of goal establishment and plans to achieve those goals | | **Flow activity**: Go through and revise the previous program activities and exercises via a snowball effect  **Central activity**: Personal action plan: Establish the steps needed to reach their best selves  **Group discussion**: Share the action plan with the class group  **Closing**: Remind students of how all the activities and exercises covered throughout the program helped increase their well-being. Encourage them to keep on practicing and completing all their work. |

*Figure S1.* Moderated network models for Time 1, 2 and 3, separately presented by groups

*Note*. Emo = Emotional well-being; Soc = Social well-being; Psy = Psychological well-being; Dep = Depression; Anx = Anxiety; Str = Stress.


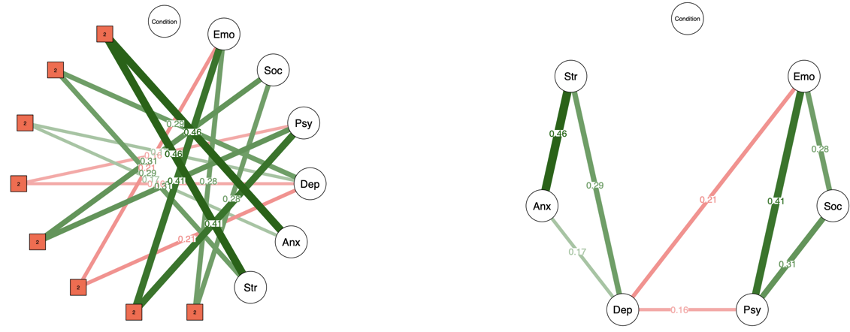


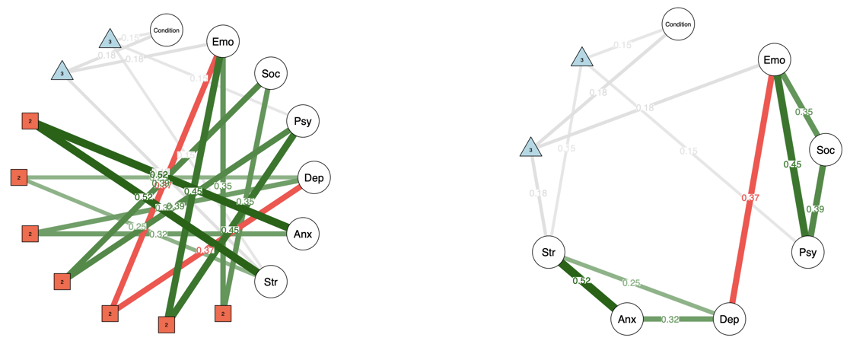


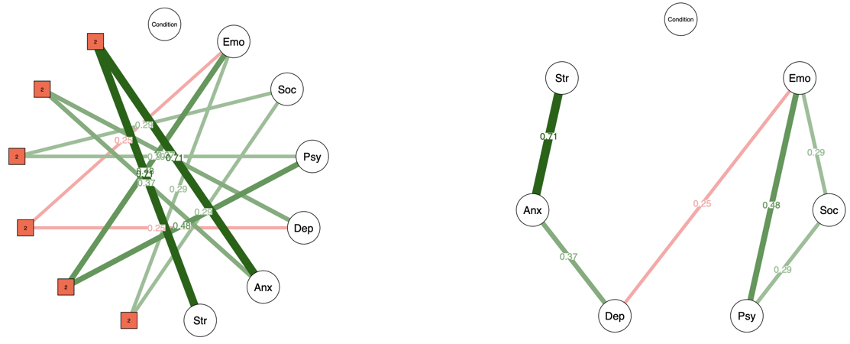


*Figure S2.* Factor graphs of the MNM at Time 1, 2 and 3, respectively. Factor graphs allow a powerful visualization of moderated networks by including 3-way and 2-way interactions as additional nodes. In the left panel, white nodes represent the network variables, red squares represent pairwise interactions, and blue triangles represent 3-way interactions (moderations). In the right panel, 3-way interactions (moderations) are presented as factor nodes and pairwise interactions as edges between variables. Blue edges represent positive linear relationships, red edges represent negative linear relationships, and grey edges represent relationships related to the moderator.

*Note*. Abbreviations are described in Figure S1. Condition is the grouping variable (control or experimental group).


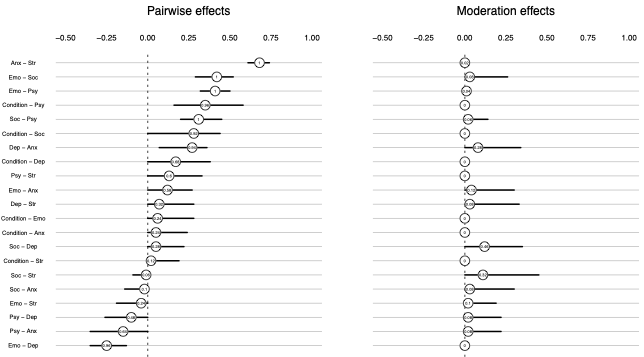


*Figure S3.* Bootstrapped sampling distributions of the simulated null model (no moderation effects).

*Note*. Abbreviations are described in Figure S1. Condition is the grouping variable (control or experimental group).
